# Retraction Note: The profile of the key pro-inflammatory cytokines in the serum of patients with CD and their association with the disease severity and activity

**DOI:** 10.1186/s12876-023-02673-y

**Published:** 2023-03-02

**Authors:** Ahmed Al Qteishat, Kiril Kirov, Dmitry Bokov

**Affiliations:** 1 Department of Biological Sciences and Chemistry, University of Nizwa, PC 616, Birkat Al-Mouz, 33, Nizwa, Sultanate of Oman; 2 Research Institute, Medical University Pleven, Sv. Kliment Ohridski Str., 1, 5800 Pleven, Bulgaria; 3 Institute of Pharmacy, Sechenov First Moscow State Medical University, Trubetskaya Str., 8/2, Moscow, Russian Federation 119991; 4 Laboratory of Food Chemistry, Federal Research Center of Nutrition, Biotechnology and Food Safety, Moscow, Russian Federation

**Retraction Note to: BMC Gastroenterology (2022) 22:477** **https://doi.org/10.1186/s12876-022-02562-w**

The Editor has retracted this article. The authors have been unable to provide evidence that ethics approval had been obtained prior to the commencement of the study. In addition, an investigation by the publisher found evidence to suggest that authorship for this article was offered for sale before the article was submitted to the journal. Therefore the Editor-in-Chief has lost confidence in the integrity of this article. Kiril Kirov and Dmitry Bokov disagree to this retraction. Ahmed Al Qteishat has not responded to any correspondence from the Editor about this retraction.

